# On-screen image-guided lead placement in cardiac resynchronization therapy: Feasibility and outcome in a multicenter setting

**DOI:** 10.1016/j.hroo.2022.10.002

**Published:** 2022-10-18

**Authors:** Philippe C. Wouters, Frebus J. van Slochteren, Anton E. Tuinenburg, Pieter A. Doevendans, Maarten-Jan M. Cramer, Peter-Paul H.M. Delnoy, Vincent F. van Dijk, Mathias Meine

**Affiliations:** ∗ Department of Cardiology, UMC Utrecht, Utrecht, the Netherlands; † CART-Tech BV, Utrecht, the Netherlands; ‡ Netherlands Heart Institute, Utrecht, the Netherlands; § Department of Cardiology, Isala Hospital, Zwolle, the Netherlands; ¶ Department of Cardiology, St Antonius Hospital, Nieuwegein, the Netherlands

**Keywords:** Cardiac resynchronization therapy, Heart failure, Magnetic resonance imaging, Image guidance, Image overlay

## Abstract

**Background:**

Image guidance to assist left ventricular (LV) lead placement may improve outcome after cardiac resynchronization therapy (CRT), but previous approaches and results varied greatly, and multicenter feasibility is lacking altogether.

**Objective:**

We sought to investigate the multicenter feasibility of image guidance for periprocedural assistance of LV lead placement for CRT.

**Methods:**

In 30 patients from 3 hospitals, cardiac magnetic resonance imaging was performed within 3 months prior to CRT to identify myocardial scar and late mechanical activation (LMA). LMA was determined using radial strain, plotted over time. Segments without scar but clear LMA were classified as optimal for LV lead placement, according to an accurate 36-segment model of the whole heart. LV leads were navigated using image overlay with periprocedural fluoroscopy. After 6 months, volumetric response and super-response were defined as ≥15% or ≥30% reduction in LV end-systolic volume, respectively.

**Results:**

Periprocedural image guidance was successfully performed in all CRT patients (age 66 ± 10 years; 59% men, 62% with nonischemic cardiomyopathy, 69% with left bundle branch block). LV leads were placed as follows: within (14%), adjacent (62%), or remote (24%) from the predefined target. According to the conventional 18-segment model, a remote position occurred only once (3%). On average, 86% of patients demonstrated a volumetric response (mean LV end-systolic volume reduction 36 ± 29%), and 66% of all patients were super-responders.

**Conclusion:**

On-screen image guidance for LV lead placement in CRT was feasible in a multicenter setting. Efficacy will be further investigated in the randomized controlled ADVISE (Advanced Image Supported Lead Placement in Cardiac Resynchronization Therapy) trial (NCT05053568).

## Graphical abstract

**Figure:**
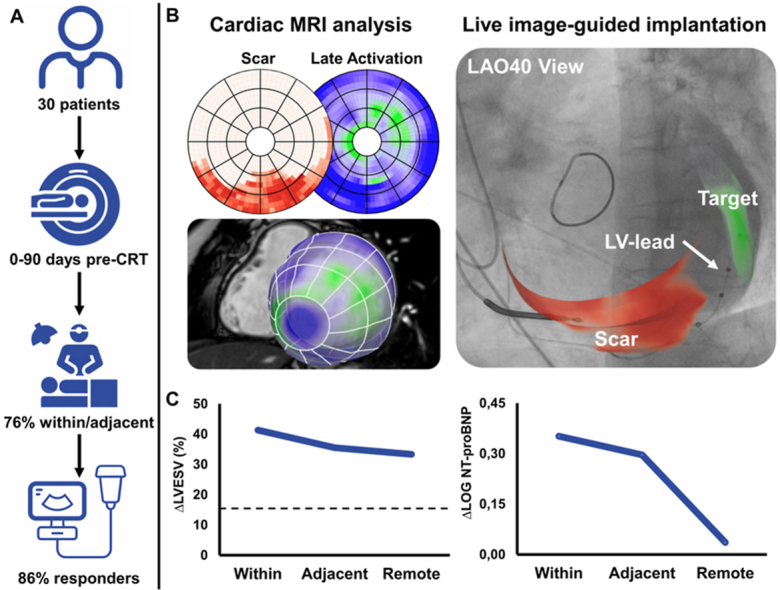

**Key Findings**

▪ For the first time, feasibility of live image guidance for cardiac resynchronization therapy was demonstrated in a multicenter setting.
▪ Fusion of cardiac magnetic resonance with dual-view fluoroscopic venograms allowed for accurate guidance and easy clinical adoption.
▪ Echocardiographic response was 86%, with 76% of leads positioned in close proximity to the patient-specific optimal target.
▪ Accurate segmental analysis and time-dependent visualization of radial strain may have contributed to our results.

Over the years, various approaches have been studied to prevent nonresponse after cardiac resynchronization therapy (CRT), or further improve treatment efficacy in patients already demonstrating a response.^1^ To this end, optimizing left ventricular lead placement (LVLP) remains crucial, as optimal device programming cannot overcome a suboptimal position.^2^ Regardless, the process of LVLP itself has remained largely unaltered, as LV leads are still routinely placed empirically.^3^

It has been shown previously that placing the lead remote from scar and within late electromechanically activated segments improves response.^4^^,^^5^ The optimal LV lead location is therefore highly variable and patient-specific.^2^^,^^6^ Although initial prospective studies were encouraging,^4^^,^^5^ feasibility of in-target LVLP, efficacy, and methodology varied largely.^6^^,^^7^ In addition, most studies were performed in a single-center setting.^6^^,^^7^ Despite its potential benefits, further development of a guided patient-tailored approach in everyday practice is still lacking.

Because of inconsistent results, the optimal strategy for LVLP is still debated.^3^ In contrast to echocardiography, cardiac magnetic resonance (CMR) imaging does not suffer from high user dependence or poor acoustic windows. In addition, CMR allows for whole heart analysis and is not confined to a limited amount of segments of the LV lateral wall.^8^ Moreover, CMR is characterized by excellent spatial resolution, without the need for ionizing radiation, and is the gold standard technique for the identification of scar and myocardial viability.^9^

The present study therefore set out to study the feasibility, and preliminary efficacy, of a dedicated device for image-guided LVLP in a multicenter setting.

## Methods

### Study design

We prospectively included 30 consecutive patients from 3 participating centers. Patients with a class I and class IIa guideline indication for a de novo CRT implantation were eligible.^3^ Exclusion criteria were impediments for CMR (ie, claustrophobia or contrast allergy) and permanent atrial fibrillation. Ischemic cardiomyopathy (ICM) was defined using clinical history or presence of ≥5% of the LV myocardial volume on late gadolinium enhancement (LGE) CMR being scar. LV ejection fraction (LVEF) and cardiac dimensions were calculated using Simpson's modified biplane method. The primary endpoint was reduction of LV end-systolic volume (ΔLVESV) 6 months after CRT implantation. Volumetric response was defined as LVESV reduction ≥15%, whereas super-response was defined as decrease of LVESV ≥30%. In addition, at 2 (interquartile range [IQR] 2–3) months, absolute reduction in log-transformed N-terminal pro–brain natriuretic peptide (NT-proBNP) was calculated. The trial was registered at the Netherlands Trial Register (trial NL8506) and approved by the Medical Research Ethics Committee Utrecht. All patients gave written informed consent. The research reported in this paper adhered to the Helsinki Declaration guidelines.

### Image acquisition and processing

**Figure 1:**
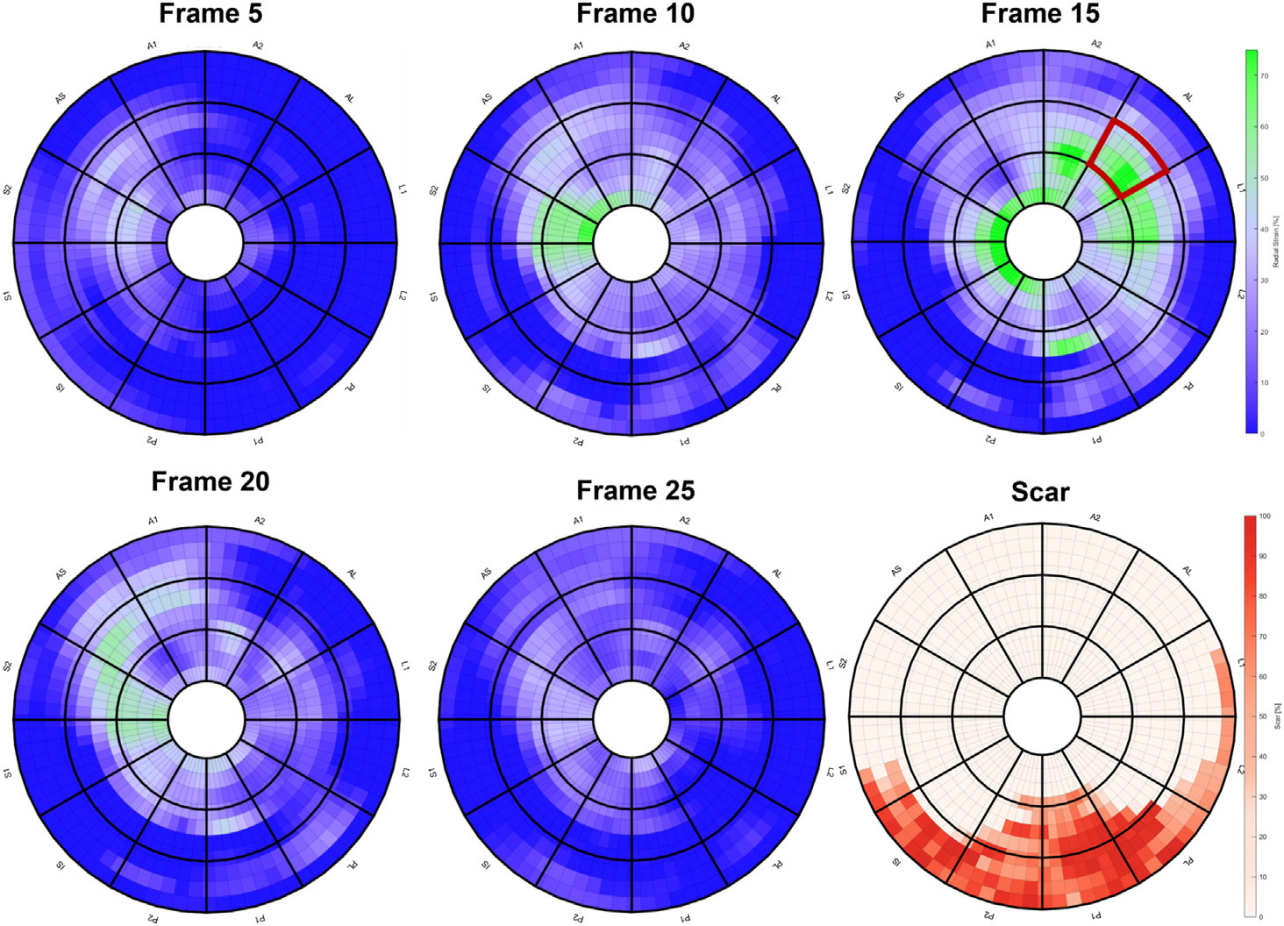
Mechanical activation starts early at the septum (frame 5) and progresses heterogeneously toward the mid-anterolateral region of the left ventricle lateral wall (frame 15). Conversely, the posterior and posterolateral wall show scar.

Within 3 months before CRT implantation, standard clinical cine-CMR with LGE sequences (1 [IQR 0–2] month) were acquired, as previously described.^10^ Short-axis CMR images were used to determine LV lead targets. Identification of LV lead targets was done using a software toolbox (CARTBox-Suite V3.1, CARTTech BV, Utrecht, the Netherlands). Myocardial scar was assessed by applying a full width at half maximum algorithm on LGE scans (Figure 1). This algorithm identifies the highest-intensity pixel within the myocardium and sets a lower threshold at half its maximum value. All pixels with values above this threshold are identified as scar. To fine-tune the scar segmentation the user can adjust the threshold and manually erase pixels to exclude small areas of blood that are erroneously segmented. Interobserver agreement concerning the location of scarred segments was strong (Cohen’s κ = 0.866; *P <* .05). For late mechanical activation (LMA), feature tracking postprocessing of the cine sequences was used to determine myocardial deformation in the radial direction. As a means of enhancing interpretability and improving signal-to-noise ratio, the magnitude of radial deformation was calculated and projected, over time, on geometrical 2-dimensional (2D) and 3-dimensional (3D) cardiac models. This resulted in 4-dimensional mechanical activation plots (4D-MAPs), in which the radial strain amplitude is displayed over time. Sites of LMA were identified as having the latest high radial strain amplitude, as displayed in a patient-specific fashion (Figure 1; Video 1).

### LV lead target specification

Earlier image-guided LVLP studies used the 16-segment American Heart Association models.^11^, ^12^, ^13^ By contrast, we incorporated a more specific 36-segment model, which allowed us to differentiate between more segments deemed relevant for LVLP. A predefined decision model was used to assist clinical decision making and improve reproducibility and user independence of target selection for LVLP. To this end, segments with myocardial scar were avoided at all times. In total, 3 sites with clear LMA, as evidenced on the 4D-MAP, were selected. The primary predefined site was the site with the most pronounced activation delay. This site was targeted and used as reference for statistical analyses (ie, within, adjacent, or remote). Apical segments were analyzed, but the apical cap was excluded as a potential LV lead target. Ultimately, the lead was navigated to the primary target (ie, LMA but no scar). The electrode closest to the predefined target was selected as the pacing electrode, in case of acceptable stimulation thresholds and absence of phrenic nerve stimulation.

### Image overlay using model-to-image registration

The preprocedural CMR-derived 3D LV surface models were superimposed on live fluoroscopic imaging (ie, model-to-image fusion). To this end, both a 3D and 2D technique can be used. Because 3D methods impose excessive radiation burden and are not available in all operating theaters, an easy-to-use 2D image registration technique to register the 3D LV surface model was developed. The 3D and 2D fusion techniques were evaluated for noninferiority in the first 5 patients of this study (Supplemental Table 1). Upon validation, only the 2D registration technique was applied in all subsequent patients (Supplemental Figure 1).

**Figure 2:**
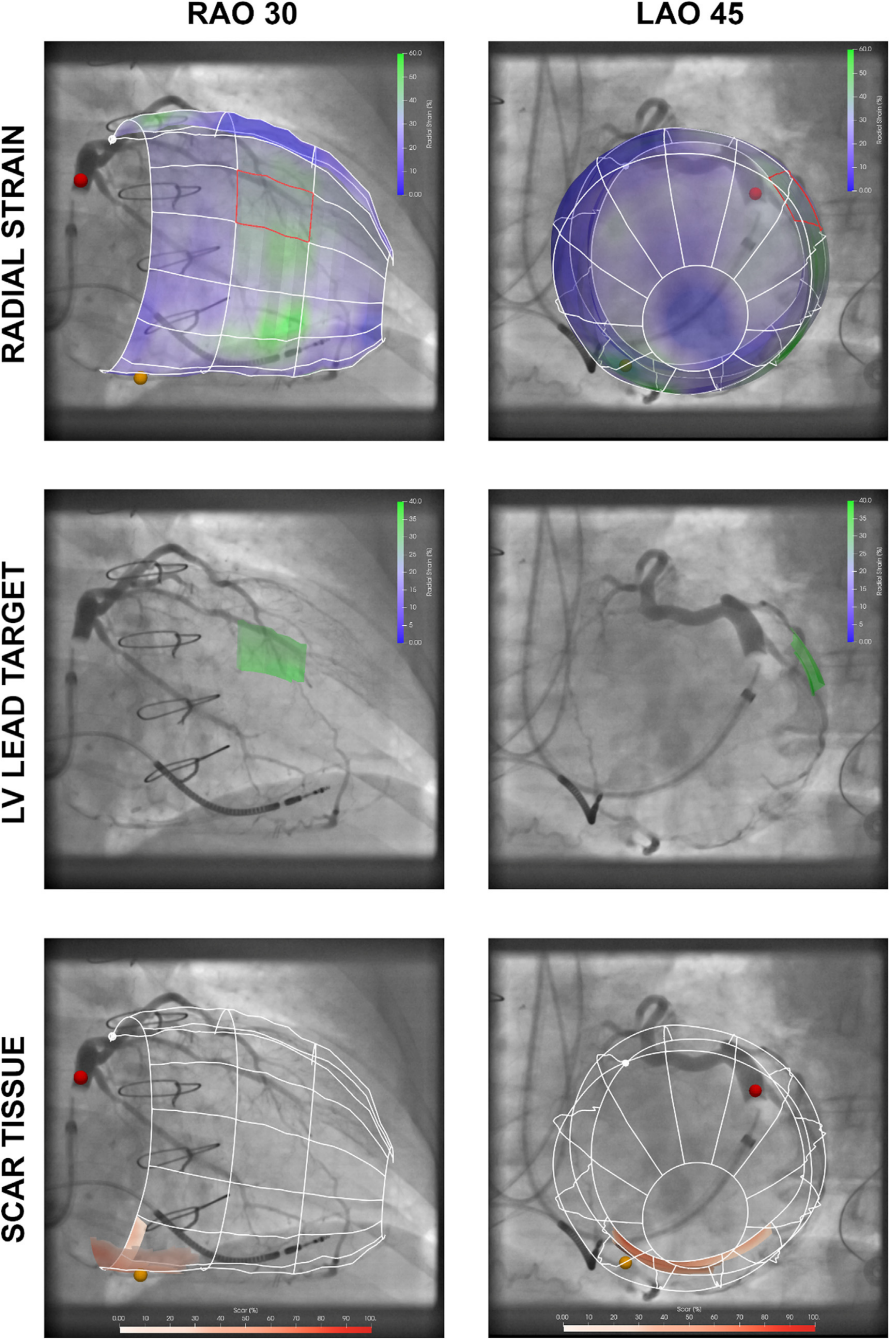
Model-to-image registration to guide left ventricular (LV) lead implantation in real time. The 36-segment mesh is derived from cardiac magnetic resonance imaging and is superimposed on dual-view fluoroscopic venograms, using the right anterior oblique (RAO) (left column) and left anterior oblique (LAO) (right column) acquisitions. Sites of latest mechanical activation (green, upper panels) are targeted and scar tissue (red, lower panels) is avoided. In this case, the most suitable target for lead implantation is determined as mid-lateral 1 and displayed to the implanting physician (middle panel). The red dot indicates the coronary sinus and the orange dot the middle cardiac vein.

In brief, EP Navigator (Philips Healthcare, Best, the Netherlands) and CART-Box Suite Light (CART-Tech BV) were used. Periprocedural 3D CMR to 2D fluoroscopy registration was performed using 2 separate 2D fluoroscopic registrations. Here, 2 acquisitions of the LV and coronary venous anatomy were acquired during balloon occlusion and contrast infusion, using an offset of at least 60 degrees (typically, left anterior oblique 40 and right anterior oblique 30). Image fusion was performed using anatomical landmarks (Figure 2). The image overlay, containing scar and the LMA target area, was superimposed on fluoroscopic images during the implantation procedure, aimed at increasing spatial lead-to-target proximity (Figure 2).

### CRT implantation and electrical measurements

Implantations were performed according to local protocols, always using quadripolar leads (Supplemental Appendix). There was no multipoint pacing. Although specific atrioventricular and interventricular optimization algorithms were rarely used, this decision was left at operator discretion. Electrical activation delay was measured, defined as the intrinsic interval between onset of the QRS complex on the ECG and local LV sensing delay on the intracardiac electrogram at a given LV pacing site (Q-LVsense). At the end of the procedure, implanting physicians were asked to briefly self-evaluate whether image guidance affected their approach.

### Allocation of final lead position

Final LV lead positions, relative to the predefined target, were determined at the end of the study by 2 observers blinded to targets, patient characteristics, and outcome. Because defining final LV lead position is unreliable using fluoroscopy only,^14^ the same model-to-image registration approach on 2D fluoroscopic images was performed. Only slight agreement was found when comparing LVLP based on fluoroscopy only^15^ with model-to-image registration (Cohen’s κ = 0.078; *P =* .536), confirming the necessity of the latter registration technique.

### Statistical analysis

Statistics were performed in SPSS version 26 (IBM, Armonk, NY). Depending on normal distribution, continuous data were expressed using mean ± SD or as median and IQR. Categorical data were expressed as the absolute number of occurrences and associated frequency. Independent subgroups were compared using a *t* test or Mann-Whitney *U* test, where appropriate. Fisher's exact test was used to compare nominal variables. One-way analysis of variance was used to compare 3 categories of lead locations. Interobserver reliability was determined using the intraclass correlation coefficient, or Cohen’s κ for categorical variables. All statistical tests performed were 2-tailed, and a *P* value <.05 was considered statistically significant.

## Results

A total of 87 de novo CRT patients were screened. Four were excluded because a contraindication for CMR (all claustrophobia) and 53 because of no consent, permanent atrial fibrillation, or no available CMR. Thirty consecutive patients were included and underwent CRT implantation between December 2019 and May 2021. One patient had no suitable venous anatomy and required epicardial lead placement, resulting in exclusion from further analysis. All 29 remaining patients successfully underwent periprocedural image guidance (Table 1). Scar burden, relative to the whole LV, was ≥5% in 10 patients (median 7.75% [IQR 5.2%–13.7%]).

**Table 1:** Baseline clinical characteristics of study population

	Total (N = 29)	Responders (n = 25)	Nonresponders (n = 4)	*P* value
Clinical characteristics				
Male	17 (59)	13 (52)	4 (100)	.121
Age, y	66 ± 10	66 ± 11	64 ± 9	.766
Non-ICM	18 (62)	17 (68)	1 (25)	.107
NYHA functional class II	16 (55)	13 (52)	3 (75)	.606
NT-proBNP, pg/mL	1529 ± 1580	1494 ± 1596	1897 ± 1917	.739
History of AF	5 (17)	4 (16)	1 (25)	.553
Type 2 DM	5 (17)	5 (20)	0 (0)	1.000
Electrocardiographic parameters				
LBBB∗	20 (69)	19 (76)	1 (25)	.076
QRS duration, ms	169 ± 20	170 ± 19	159 ± 23	.285
Medication				
β-blocker	23 (79)	21 (84)	2 (50)	.180
ACE inhibitor/ARB	27 (93)	23 (92)	4 (100)	1.000
Spironolactone	12 (41)	9 (36)	3 (75)	.279
Echocardiographic parameters				
LVEDV, mL	209 ± 79	212 ± 85	190 ± 12	.604
LVESV, mL	163 ± 68	166 ± 72	141 ± 15	.503
LVEF, %	23 ± 7	22 ± 7	26 ± 4	.310
TAPSE, mm	18 ± 5	18 ± 5	21 ± 5	.192

Values are n (%) or mean ± SD.

ACE = angiotensin-converting-enzyme; AF = atrial fibrillation; ARB = angiotensin II receptor blocker; DM = diabetes mellitus; ICM = ischemic cardiomyopathy; LBBB = left bundle branch block; LVEDV = left ventricular end-diastolic volume; LVEF = left ventricular ejection fraction; LVESV = left ventricular end-systolic volume; NT-proBNP = N-terminal pro–brain natriuretic peptide; NYHA = New York Heart Association; TAPSE = tricuspid annular plane systolic excursion.

∗ According to the European Society of Cardiology definition.

### Multicenter feasibility of periprocedural image fusion

Complete CMR analysis was completed within 2 days after receiving data from participating hospitals. Image fusion at the catheter laboratory was performed in 22 ± 7 minutes for 3D fusion and within 5 minutes for 2D fusion. According to operating physicians, image fusion was displayed on time in 28 (96%) cases, thereby not significantly postponing LVLP. Average duration of LVLP was 50 ± 35 minutes, with a total procedure time of 120 ± 45 minutes. On average, 55 ± 28 mL of contrast fluid was used. Radiation dose area was lower using the 2D fusion method as compared with using the 3D rotational fluoroscopy (2805 ± 4051 μGy/m^2^ vs 4840 ± 2625 μGy/m^2^; *P =* .299).

### LV lead positioning and target allocation

Based on the 36-segment model, interobserver comparison reproduced the exact same primary target selection in 12 (63%) cases and an adjacent one 6 (32%) times. There was substantial agreement when evaluating final LV lead location categories, based on the primary target of the 2 different observers (Cohen’s κ = 0.680; *P <* .001).

**Figure 3:**
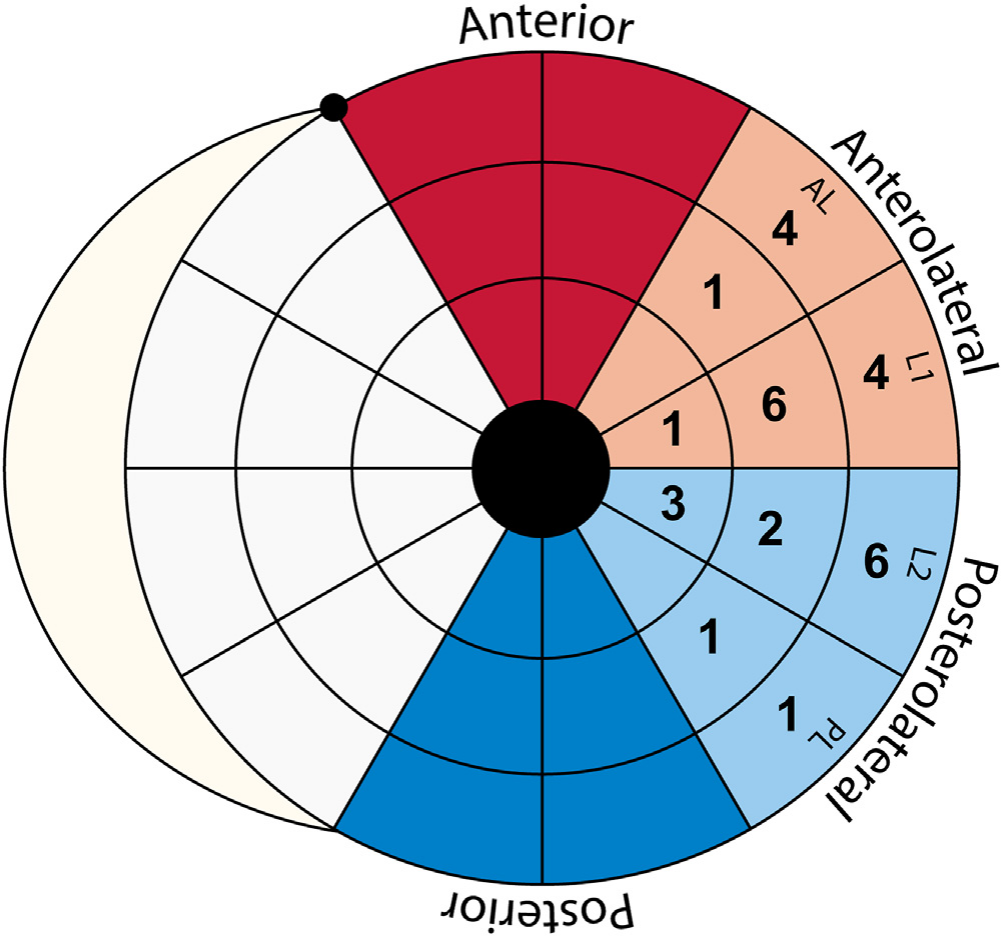
Bullseye plot according to the 36-segment model (individual parts) and traditional 18-segment model (colorized parts). Leads were distributed across 10 different locations, whereas 8 different segments were identified as optimal.

Figure 3 displays final lead distribution, with the majority of leads positioned within (n = 4 [14%]) or adjacent (n = 18 [62%}) to the predefined target. On average, midventricular segments were 4.3 ± 1.2 cm^2^ in size, whereas basal segments averaged 6.7 ± 1.5 cm^2^. According to either the 36-segment or conventional 18-segment American Heart Association model, a remote position was acquired in 24% (n = 7) or 3% (n = 1) of patients, respectively. Documented explanations for deviation of the advised primary target were lack of venous access (n = 5), high pacing thresholds (n = 2), phrenic nerve stimulation (n = 1), or unstable lead position (n = 1).

### Electrical properties

Q-LVsense of the stimulation electrode (average 157 ± 35 ms) was similar when stratified to lead position (≥150 ms, regardless of vicinity to the target) and unaffected by presence of scar (Supplemental Figure 2). Intrinsic LV electrical delay, normalized to QRS duration (Q-LVsense/QRSd), was not associated with a volumetric response (area under the curve 0.542; *P =* .793). When separated at the median, high vs low Q-LVsense/QRSd was not associated with ΔLVESV (37 ± 28% vs 32 ± 28%; *P =* .572). Pacing thresholds at the final electrode were 0.9 ± 0.5 V. At 2-month and 6-month follow-up, the 2-month biventricular pacing percentage was on average 95 ± 13% (<95% in 4 patients). No LV pacing vectors were altered and no shocks were delivered.

### Volumetric and neurohumoral response

**Figure 4:**
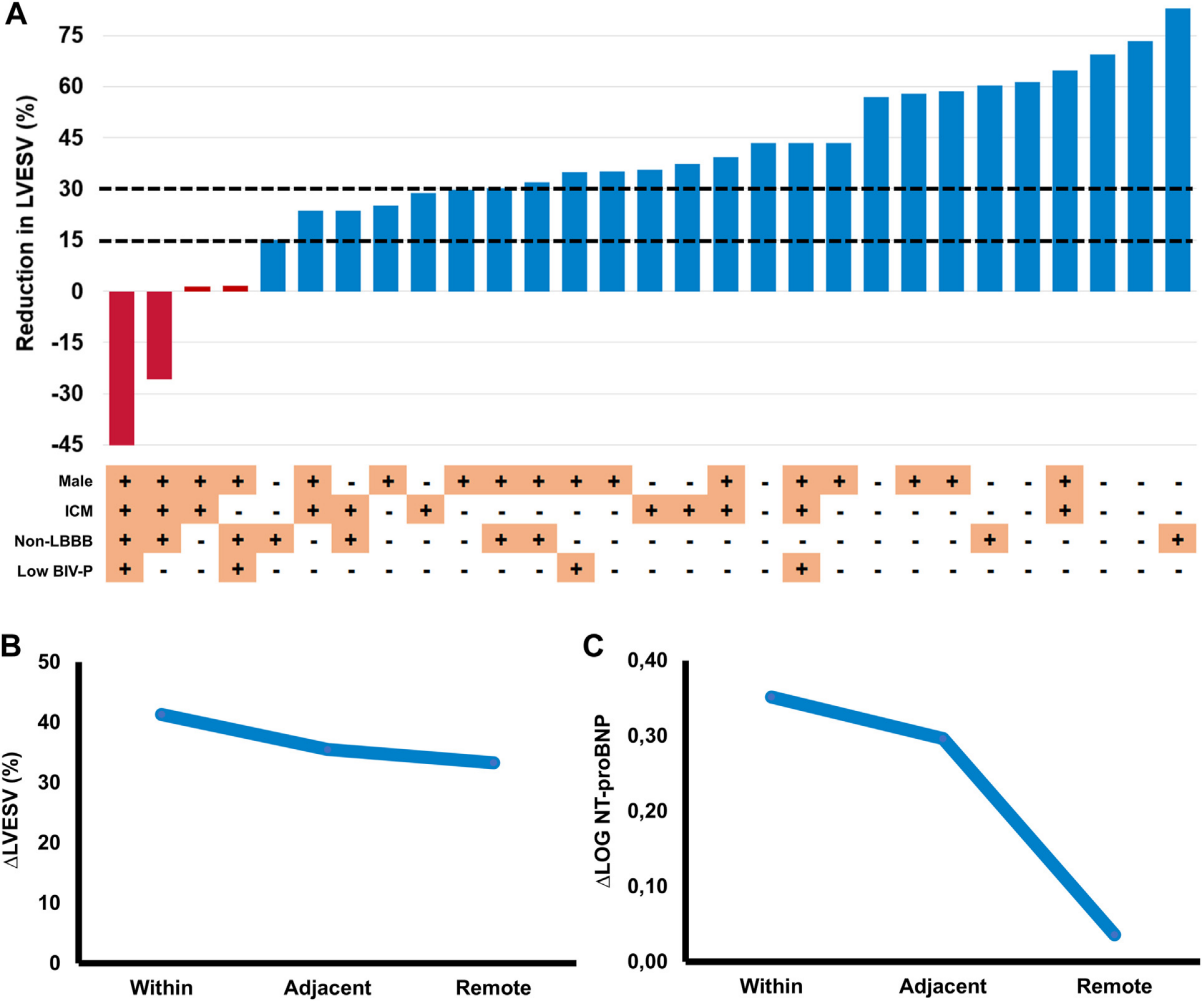
Volumetric and neurohumoral response. **A:** In total, 86% of patients were volumetric responders. **B, C:** Although change in left ventricular end-systolic volume (ΔLVESV) was not significantly related to lead position, an association with log-reduction in N-terminal pro–brain natriuretic peptide (NT-proBNP) was observed. BIV-P = biventricular pacing <95%; ICM = ischemic cardiomyopathy; LBBB = left bundle branch block.

In total, average reduction in LVESV was 36 ± 29%, with 25 (86%) patients being volumetric responders and 19 (66%) super-responders (Figure 4A). Volumetric response rates were 95% in non-ICM, 94% in left bundle branch block (LBBB), 73% in ICM, and 67% in non-LBBB. Although not significant, the 4 nonresponders were more frequently characterized by ICM, non-LBBB, and not being on β-blockers (Table 1). In-scar pacing was unavoidable in 2 cases, which were both nonresponders with a mean increase in LVESV of 35 ± 14% (Supplemental Figure 2). Excluding in-scar pacing, LVESV reduction in patients with and without ICM (33 ± 17% vs 45 ± 21%; *P =* .158) or non-LBBB and LBBB (35 ± 28% vs 43 ± 18%; *P =* .380) was nonsignificantly different.

Volumetric response was comparable between different anatomical LV lead locations (Figure 4B). A nonremote lead position resulted in similar reverse remodeling when compared with remote lead placement (37 ± 25% vs 33 ± 38%; *P =* .796). Absolute LVEF increased on average by 13 ± 12%, with 45% of patients having their LVEF increased to ≥35% (Table 2). Interobserver reliability for echocardiographic measurements of LVESV was excellent (intraclass correlation coefficient = 0.990 [95% confidence interval 0.956–0.998]; *P <* .001). Log-transformed NT-proBNP decreased significantly at 2-month follow-up (3.03 vs 2.82; *P =* .013), with a nonsignificant trend between NT-proBNP and lead position (η^2^ = 0.220; *P =* .199) (Figure 4C).

**Table 2:** Changes in echocardiographic function

Variable	Baseline	6 mo	*P* value
LVEDV, mL	209 ± 79	153 ± 68	<.001
LVESV, mL	163 ± 68	101 ± 55	<.001
LVEF, %	22 ± 7	35 ± 12	<.001
IVMD, ms	87 ± 85	34 ± 31	.002
TAPSE, mm	18 ± 5	18 ± 7	.759
RVS′, cm/s	10 ± 3	12 ± 4	.036

Values are mean ± SD.

IVMD = interventricular mechanical delay; LVEDV = left ventricular end-diastolic volume; LVEF = left ventricular ejection fraction; LVESV = left ventricular end-systolic volume; RVS′ = right ventricular systolic velocity; TAPSE = tricuspid annular plane systolic excursion.

### Influence of image guidance on decision making

Before image guidance, the brief user questionnaire revealed a mid-(antero)lateral position as most frequently preferred target by the physician. The 4D-MAP analysis identified a target adjacent or remote from the physicians’ target in 39% and 33% of cases, respectively. Implanting physicians noted that image guidance significantly altered the implantation by navigating toward another target in 38% of cases. In 19% of all cases, another vein was chosen. They noted that the procedure was either somewhat prolonged or shortened in 21.7% and 13.0%, respectively. Ultimately, image guidance was perceived as helpful in 60.7% of cases.

## Discussion

We demonstrate the use of image-guided LVLP in a multicenter setting, using an accurate 36-segment model. Within- or adjacent-from-target LVLP occurred in 76% of cases, with 86% of patients classified as responders. However, according to the 18-segment model, only 1 patient had the LV lead implanted in a truly remote position. Model-to-image fusion, combining CMR and live dual-view fluoroscopic venograms, is therefore feasible in a multicenter setting.

### Determinants of response to CRT

There were 4 patients in our series who did not respond to CRT. Although patient characteristics also determine response to CRT, a poor LV lead position within scar likely explained nonresponse in 2 of 4 nonresponders (Supplemental Figure 2). Importantly, if in-scar pacing could be avoided, despite a clear scar burden, response rates were high in patients with ICM and non-LBBB. Conversely, optimal pacing areas are relatively larger in patients with LBBB and non-ICM,^6^ and the majority of leads were optimally placed, which may explain why no clear association was found between lead-to-target proximity and response.

### Periprocedural image-guided lead implantation

One previous study required CMR acquisition and CRT implantation in a single session, complicating its use in clinical practice.^11^ Two other studies targeted segments with the most delayed time-to-minimum volume, using regional volume-over-time curves.^11^^,^^12^ By contrast, our approach used time-dependent visualization of mechanical activation. This likely reduced the influence of noise and improved interpretability relative to conventional approaches, as underscored by the substantial interobserver agreement for target selection in the present study. To our knowledge, previous studies did not report reproducibility of target selection. Last, traditional larger-segment models less accurately portray spatial lead-to-target proximity, which limits how precise regions with scar and LMA can be visualized and targeted.

### Feasibility in multicenter setting

Only 34% of the 87 screened de novo CRT patients were enrolled, in part because timely acquisition of a new CMR was not always possible. However, actual contraindications for CMR were rarely encountered. Regardless, image guidance was successfully performed in all CRT patients. Notwithstanding limited venous access or high pacing thresholds, which precluded optimal LVLP in some patients, a near-optimal position was acquired in the majority of cases. In addition, implantation times compared favorably with all previously conducted live image-guided studies.^11^, ^12^, ^13^ When comparing differences in spatial lead-to-target proximity, adjacent LVLP, according to 36 segments, can be considered similar to within target in the 18-segment model (Figure 2). Unfortunately, feasibility of previous live image-guided approaches was tested in single-center settings only, and with smaller sample sizes.^11^, ^12^, ^13^ Yet, the percentage of 6-month volumetric responders was lower, at 60%,^12^^,^^13^ or not investigated.^11^ Last, these studies enrolled more patients with LBBB^11^, ^12^, ^13^ and included comparable amounts of patients with ICM.^12^^,^^13^

### Electrical guiding as alternative

Maximizing the Q-LVsense interval is a well-recognized strategy to enhance response to CRT. Although Q-LVsense is associated with CRT response on the group level,^7^ it cannot differentiate optimal from suboptimal segments in individual patients.^16^ Differentiation is especially difficult when Q-LVsense is high, or when differences in Q-LVsense at various locations of a quadripolar lead are small, which is the case when leads are already placed in or near an optimal location.^6^^,^^16^ Hence, lack of association between Q-LVsense and remodeling is also reflected by our results, as average Q-LVsense at the stimulation electrode was ≥150 ms, and similar across patients with different LV lead locations. Mapping Q-LVsense in all suitable epicardial veins may prove more effective, but this approach is time-consuming and cumbersome.^17^ Moreover, Q-LVsense guidance provides no additional benefit in patients with non-LBBB.^18^ By contrast, image guidance has shown to be beneficial in this subgroup and can preprocedurally characterize the mechanical delays of the whole LV lateral wall.^4^

### Clinical relevance and outlook

In theory, image guidance may be most valuable in patients with non-LBBB and ICM, as these patients typically demonstrate heterogeneous LV electrical activation, have smaller target sites, and demonstrate poorer outcome after CRT when compared with non-ICM or LBBB patients.^6^ Although CMR is costly, an increase of 5% in the proportion of CRT responders may render an image-guided technique cost-effective.^19^ Moreover, relative to approaches that lack fluoroscopic overlay,^6^ use of periprocedural image assistance substantially increase the in-target success rate from 30% to 63% to from 71% to 83%.^11^, ^12^, ^13^ These findings are in line with our results, confirming the importance of model-to-image registration to achieve in-target LVLP. Moreover, using an image-guided approach to determine the optimal pacing site provides a faster and less invasive alternative to using acute hemodynamic measurements.^20^

### Limitations

Although promising, our results should be interpreted with caution in the context of a nonrandomized design. The separately conducted randomized multicenter ADVISE (Advanced Image Supported Lead Placement in Cardiac Resynchronization Therapy) trial (NCT05053568) will address this important limitation.^19^ Although the high proportion of LBBB may have influenced our results, patient characteristics were comparable to previous live image-guided studies.^11^, ^12^, ^13^ Descriptive subgroup analyses were provided for hypothesis-generating purposes, but analyses were underpowered due to limited sample size. As a result, the effect of LV lead location and response warrants further research. Although CMR has high spatial resolution, better temporal resolution is achieved using speckle-tracking echocardiography. Alternatively, cardiac computed tomography may be more suitable in patients with a pre-existing ICD implanted, and can also assess venous anatomy.^13^ Last, accurate identification of LVLP using fluoroscopy is not without its pitfalls due to variable cardiac anatomy and high observer-dependent interpretation.^14^ However, our model-to-image registration approach likely reduced the risk of misclassification, without the need for post-CRT computed tomography.

## Conclusion

Use of CMR as a radiation-free and noninvasive imaging technique to guide LV lead implantation is feasible in a multicenter setting, as 76% of leads were implanted in close proximity to the target, and 86% of patients demonstrated a volumetric response, with a mean reduction in LVESV of 36%. Accurate segmental analysis, time-dependent visualization of radial strain, and periprocedural image fusion may have contributed to these promising results. The randomized controlled ADVISE trial will further study the clinical efficacy following the present approach.^19^

## Data availability

Data underlying this article will be shared on reasonable request to the corresponding author.
