# Supplementary material for: On-screen image-guided lead placement in cardiac resynchronization therapy: Feasibility and outcome in a multicenter setting
Source: Heart Rhythm O2. 2022 Oct 18;4(1):9–17. doi: 10.1016/j.hroo.2022.10.002 (PMC9877392; doi:10.1016/j.hroo.2022.10.002)
Supplement: Supplemental Appendix [file mmc1.docx]

# **Real-time image-guided lead placement in cardiac resynchronisation therapy: feasibility and outcome in a multicentre setting**

**Supplemental Appendix**

**Supplemental methods 02**

**Supplemental results 07**

**Supplemental tables 09**

**Supplemental figures 10**

**Supplemental methods**

**CRT Implantation and electrical measurements**

In brief, right atrial and right ventricular leads were placed, preferably in the right atrium appendage and right ventricular septum, respectively. In all patients, a quadripolar LV lead was implanted through the coronary sinus. Using live image-overlay, the vein deemed most suitable to facilitate lead placement at the pre-defined target was attempted first. In all patients, procedure times and radiation dose were collected. In addition, pacing threshold and phrenic nerve stimulation threshold were assessed at all stimulation electrodes.

**Image overlay using model-to-image registration**

For the registration of the 3D LV surface models with live fluoroscopy (i.e., model-to-image fusion), both a 3D and 2D technique have been used. For the 3D technique, a rotational scan was acquired using interventional cone beam cardiac tomography equipment. Subsequently, a 3D DICOM file with myocardial scar and the implantation target were registered with the reconstructed 3D rotational scan, as described previously in Salden et al (1). A drawback of the 3D technique is the necessity to acquire a 3D rotational scan, solely for the purpose of registration of the 3D LV surface models, whereas the rest of the intervention is done based on 2D fluoroscopy. This technique is not available in all operating theatres, and imposes excessive radiation burden (1). To overcome this issue, an easy to use 2D image registration technique to register the 3D LV surface model was developed. This new technique was validated in the first 5 patients in this study, and upon validation the 2D registration technique was applied in the subsequent patients **(Supplemental Figure 1)**.

For the 2D registration technique, EP Navigator [Philips Healthcare, Best, The Netherlands], and CART-Box Suite Light [CART-Tech B.V., Utrecht, The Netherlands] was used. Live 3D-CMR to 2D-fluroscopy registration was performed using two separate 2D-fluoroscopic registrations. Here, two acquisitions of the LV and coronary venous anatomy were acquired during balloon occlusion and contrast infusion, using an offset of at least 60 degrees (typically, LAO40 and RAO30). Image fusion was performed using anatomical landmarks (coronary sinus, middle cardiac vein, cardiac silhouette), in line with Babic et al. **(Figure 2)** (2). In RAO30, the 3D LV surface model was oriented in the corresponding angles and the coronary sinus was aligned nearby the base of the 3D LV surface model. Subsequently the 3D middle cardiac vein marker was aligned with the corresponding 2D location. In LAO40, the 3D LV surface model was oriented in the corresponding angles and aligned with the 2D fluoroscopy based on the cardiac silhouette. The location was fine-tuned based on the 3D markers of the coronary sinus, and middle cardiac vein. After the registration step, the overlay image, containing scar and the target area information, was shown to the cardiologist by a color scale on the large display in the operating theater **(Figure 2)**.

**Echocardiographic analysis**

Echocardiograms were obtained before (0 months [IQR -2-0]) and 6 months after CRT implantation (6 months [IQR 5-6]). LVEF and cardiac dimensions were calculated using Simpson's modified biplane method (3). Interventricular mechanical delay (IVMD) was measured as the difference between left and right ventricular pre-ejection intervals, using pulsed wave Doppler. RV-function was assessed using tricuspid annular plane systolic excursion (TAPSE) using M-mode, and tissue doppler imaging-derived tricuspid lateral annular systolic velocity wave (RV S’).

**Validation of 3D-3D versus 3D-2D registration**

**Introduction of 3D-3D registration versus 3D-2D registration**

To register a volumetric MRI or CT DICOM datasets on the commercial fluoroscopy systems to perform multimodality image guided procedures special software versions of the C-arm software are required. Either 3D image registration is done based on the acquisition of a 3D dataset using the interventional cone beam CT equipment, or based on multiple 2D recordings that differ at least 60 degrees. Drawback of both techniques is that the dataset to be loaded in the C-arm software for registration is limited to the DICOM standard. The DICOM standard has limited options visualize tissue characteristics by means of colours. Moreover, the acquisition of the 3D fluoroscopy dataset is only used for the registration, whilst the rest of the intervention is done using 2D fluoroscopy. This leads to an increased radiation dose delivered to the patient and warrants the use of an alternative less demanding and less harmful registration technique.

In this supplemental data we introduce and validate an alternative 2D model to image registration technique which reduced the radiation burden for the patient and allows the visualisation of the tissue characteristics in real time during a multimodality fluoroscopy guided CRT implantation. The new 2D registration technique can be easily implemented in existing cardiac catheterization rooms and thereby maximizes the use of tissue characteristics deduced from a pre-existing volumetric MRI or CT during cardiac interventions. In this report we validate the accuracy of the 2D registration technique for use during cardiac resynchronization therapy (CRT).

**Data acquisition**

In five patients of the present study, the 3D registration technique was used to register the pre-intervention 3D DICOM file with myocardial scar and CRT target information with the reconstructed interventional 3D rotational scan as described in Salden et al. (1). During the CRT procedure 2D fluoroscopy was acquired in the LAO40 and RAO30 orientations. During the 2D acquisitions a balloon occlusion of the coronary sinus was used to visualize the coronary sinus anatomy. After the procedure both the 3D cone beam CT dataset and the 2D images were exported from the C-arm system.

**Registration process**

In an in-house developed software application, the 3D cone beam CT dataset and the pre-intervention 3D DICOM file were registered (step 1), and the 2D images of end expiratory and end diastolic venograms in the LAO40 and RAO30 orientations were rendered in their corresponding position, orientation and scaling with respect to the registered 3D datasets (step 2). Subsequently the 3D LV surface model derived from the pre-intervention 3D DICOM file was loaded into the system with an unknown offset from its original position/orientation/scaling and was thereafter manually oriented, positioned and scaled to its correct anatomical position/orientation/scaling based on the 2D images in an iterative process (step 3). When the ultimate position was achieved according to the iterative process described above the 3D LV surface model derived from the pre-intervention 3D DICOM file was loaded into the system and rendered at its original position/orientation/scaling. By subtracting the vertices of the ground truth model in the circumferential, longitudinal and radial directions the 2D registration error in the circumferential, longitudinal and radial directions can be calculated.

**Validation**

The required accuracy for the 2D registration is based on the prevention of mis targeting the LV lead caused by a registration error. To calculate the required accuracy of the 3D model to 2D image registration we assume that:

1. The LV lateral wall is divided into 6 segments (twice as much as in the standard AHA 16 segment model)
2. There are three coronary sinus branches distributed evenly over the LV free wall (one anterior, one lateral and one posterior).

A registration error of more than half a segment in the circumferential direction would cause an implantation in the wrong CS side branch. Because the size of a segment depends on the size of the heart and this is different for each patient, the maximum permissible absolute registration error therefore is patient-specific. The analysis above results in a maximum permissible registration error of a half segment corresponding to 1/24 = 4.1% of the circumference of the heart. The registration error must therefore remain within 4.1% of the circumference of the heart.

**Supplemental results**

**LV lead positioning**

Leads were either implanted in a basal segment (52%), mid-cardiac segment (34%), or an apical segment (14%). Within the circumferential orientation, leads were placed as follows according to our 36-segment model: anterior (0%), anterolateral (17%), lateral 1 (38%), lateral 2 (38%), posterolateral (7%), posterior (0%). According to the traditional AHA model [8], this corresponds to 55% anterolateral, and 45% posterolateral **(Figure 3)**.

**Validation of 3D-3D versus 3D-2D registration**

The validation of the 2D registration was performed in five patients. The results are shown in **Supplemental Table 1**. The results are a pointwise subtraction of the vertices of the ground truth LV surface model and the LV surface model that is registered based on two 2D images.

**Validation Conclusion**

The positions of 3D epicardial LV surface models that are registered by two 2D registrations, using end expiratory and end diastolic venographic images recorded at LAO40 and RAO30, have a high correspondence with the positions of 3D epicardial surface meshes after volumetric (3D) registrations. Since the level of agreement lies within 4.1% of the circumference of the heart, registration of 3D epicardial surface meshes using two 2D end expiratory and end diastolic venographic images recorded at LAO40 and RAO30 is a valid alternative for the registrations based on 3D rotational X-Ray acquisitions.

Since the acquisition of two 2D end expiratory and end diastolic venographic images recorded at LAO40 and RAO30 are standard acquisitions during CRT implantation, this omits the necessity for 3D rotational X-Ray acquisitions and thereby saves time and reduces the radiation burden for the patients, whilst information from the MRI (myocardial infarction and mechanical dyssynchrony) can be easily visualized on the epicardial LV surface model during the CRT implantation.

**Supplemental tables**

***Supplemental Table 1.*** *Summary of registration differences when comparing 3D scan versus two separate 2D fluoroscopic images.*

| **Patient** | **Threshold (4% of max circumference)** | **max circumferential error** | **max longitudinal error** | **max radial error** |
| --- | --- | --- | --- | --- |
| **1** | 10.5mm (4%) | 9.7mm (3.6%) | 9.9mm (3.7%) | 7.7mm (2.9%) |
| **2** | 11.3mm (4%) | 10.9mm (3.8%) | 9.7mm (3.4%) | 10.2mm (3.6%) |
| **3** | 9.6mm (4%) | 2.2mm (0.9%) | 1.8mm (0.7%) | 2.0mm (0.8%) |
| **4** | 9.3mm (4%) | 8.0mm (3.4%) | 7.7mm (3.3%) | 7.4mm (3.2%) |
| **5** | 9.2mm (4%) | 3.8mm (1.7%) | 1.2mm (0.53%) | 3.8mm (1.7%) |

*Thresholds of the maximum difference in registration were 4% of the circumference and length of the heart. The results are a pointwise subtraction of the ground truth mesh and the mesh that is registered based on two 2D images.*

**Supplemental figures**


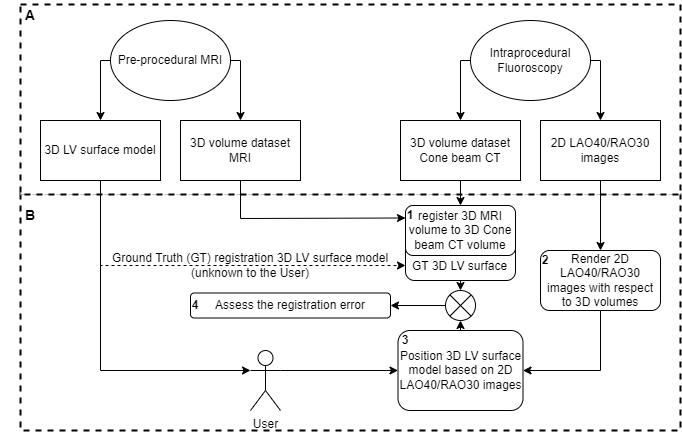


***Supplemental Figure 1:*** *Schematic overview of the registration validation process. Panel A: data acquisition. Panel B: registration steps. Step 1: Register the 3D MRI volume to the 3D Cone beam CT volumes. Step 2: render the 2D LAO40 and RAO40 images with respect to the 3D Cone beam CT volume. Step 3: Position the 3D LV surface mesh based on the anatomical landmarks (coronary sinus, middle cardiac vein, cardiac silhouette). Step 4: distract the ground truth (GT) and the registered 3D LV surface models to assess the registration accuracy.*


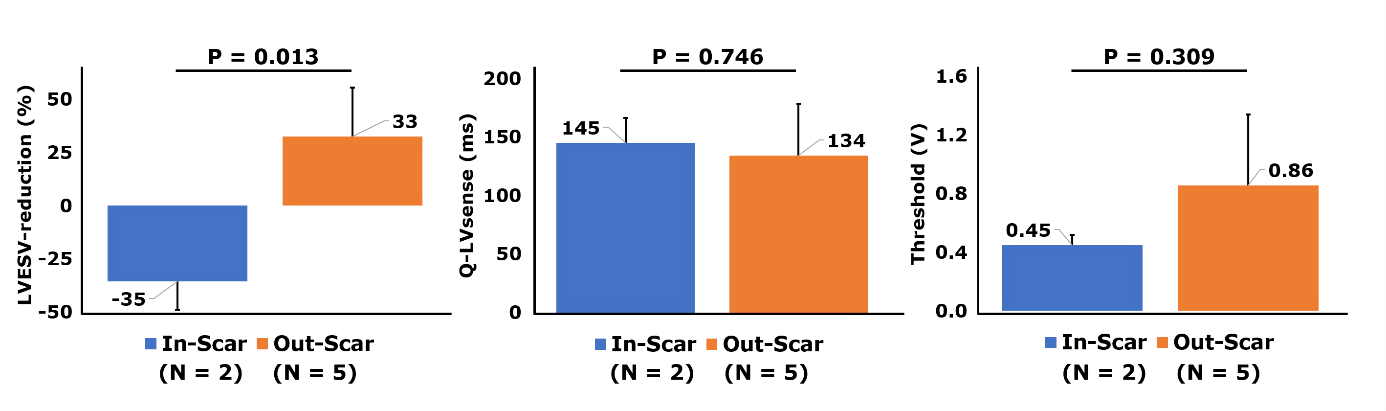


***Supplemental Figure 2.*** *In patients with ischemic cardiomyopathy and left ventricular (LV) lateral wall scar, scar at the LV pacing electrode was associated with less reverse remodelling, but electrical properties could not differentiate between in versus out-scar pacing. Legend: LVESV, left ventricular end-systolic volume.*

***Supplemental Video 1.*** *Mechanical activation starts early at the septum (frame 5), and progresses heterogeneously towards the mid-anterolateral region of the left ventricle lateral wall (frame 15).*

**Supplemental references**

1. Salden OAE, van den Broek HT, van Everdingen WM, et al.: Multimodality imaging for real-time image-guided left ventricular lead placement during cardiac resynchronization therapy implantations. Int J Cardiovasc Imaging 2019; 35:1327–1337.

2. Babić A, Odland HH, Lyseggen E, et al.: An image fusion tool for echo-guided left ventricular lead placement in cardiac resynchronization therapy: Performance and workflow integration analysis. Echocardiography, 2019; 36:1834–1845.

3. Lang RM, Badano LP, Mor-Avi V, et al.: Recommendations for cardiac chamber quantification by echocardiography in adults: an update from the American Society of Echocardiography and the European Association of Cardiovascular Imaging. Eur Hear J Cardiovasc Imaging, 2015; 28:1-39.e14.
