# Supplementary figures and images for: On-screen image-guided lead placement in cardiac resynchronization therapy: Feasibility and outcome in a multicenter setting
Source: Heart Rhythm O2. 2022 Oct 18;4(1):9–17. doi: 10.1016/j.hroo.2022.10.002 (PMC9877392; doi:10.1016/j.hroo.2022.10.002)

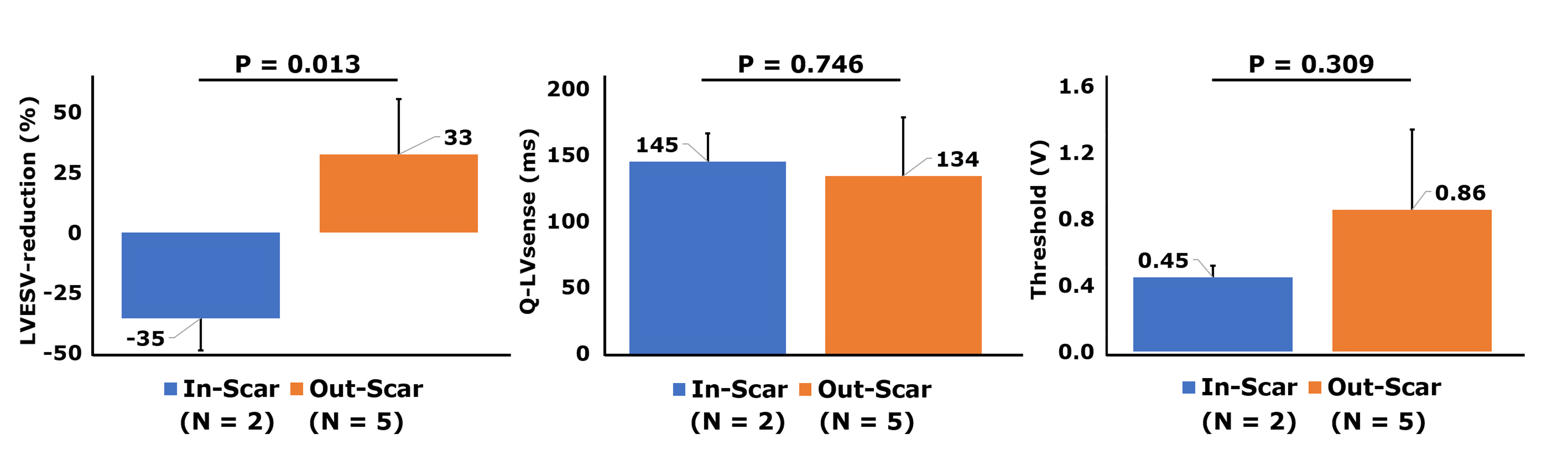

Supplement: Supplemental Figure 1 [file figs1.jpg]
